# Supplementary figures and images for: The role of the mitochondrial outer membrane protein SLC25A46 in mitochondrial fission and fusion
Source: Life Sci Alliance. 2023 Mar 28;6(6):e202301914. doi: 10.26508/lsa.202301914 (PMC10052876; doi:10.26508/lsa.202301914)

**SOURCE DATA – SDS-PAGE**

**Figure 1**

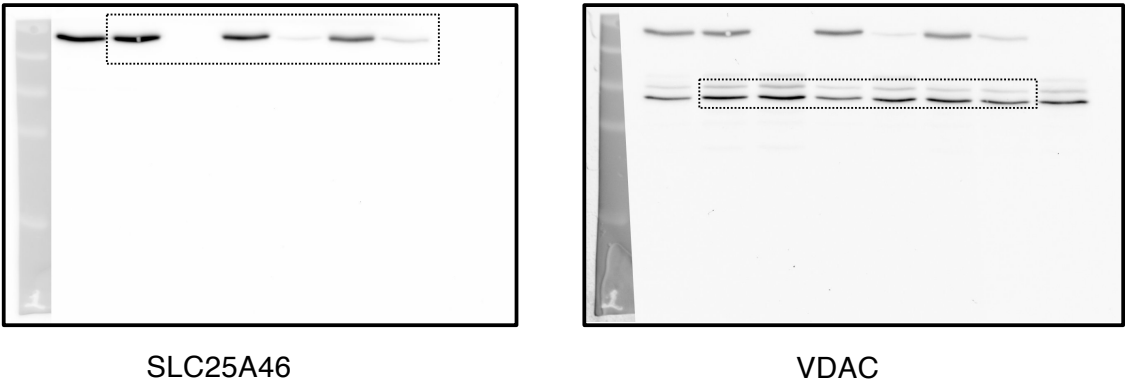

**Figure 5**

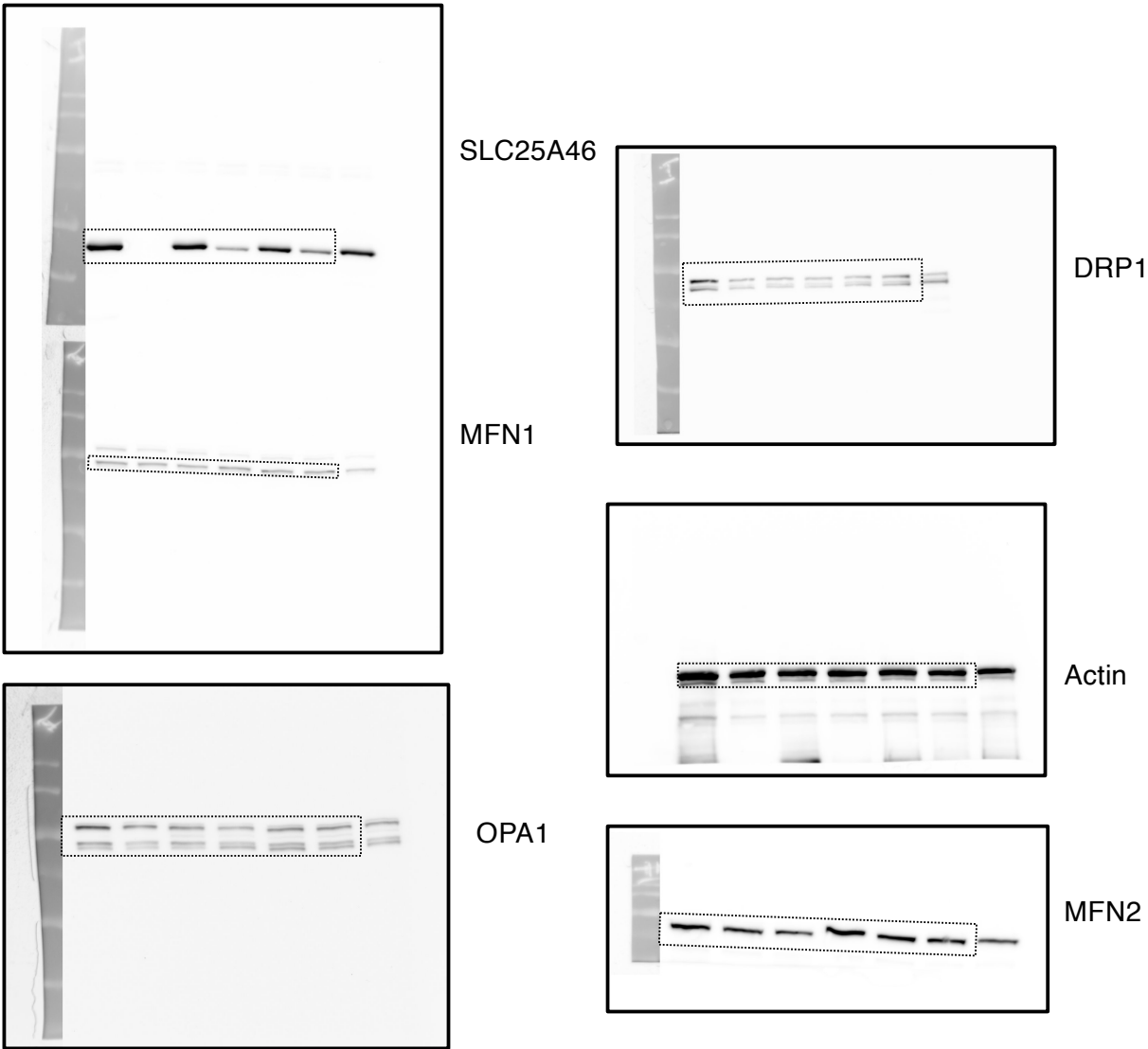

Supplement: Supplementary file 1 [file LSA-2023-01914_SdataF1_F5.pdf]
